# Supplementary material for: Anesthetic agents affect urodynamic parameters and anesthetic depth at doses necessary to facilitate preclinical testing in felines
Source: Sci Rep. 2020 Jul 9;10:11401. doi: 10.1038/s41598-020-68395-3 (PMC7347647; doi:10.1038/s41598-020-68395-3)
Supplement: Supplementary file 1 — Supplementary Information. [file 41598_2020_68395_MOESM1_ESM.pdf]

**Supplementary Information**

**Title**: Anesthetic agents affect urodynamic parameters and anesthetic depth at doses necessary to facilitate preclinical testing in felines

**Authors**: Jiajie Jessica Xu[1,2], Zuha Yousuf[2,3], Zhonghua Ouyang[2,3], Eric Kennedy[2,3], Patrick A. Lester[1], Tara Martin[1], *Tim M. Bruns[2,3]

**Affiliations**:

1. Unit for Laboratory Animal Medicine, University of Michigan, Ann Arbor, MI
2. Biointerfaces Institute, University of Michigan, Ann Arbor, MI
3. Biomedical Engineering Department, University of Michigan, Ann Arbor, MI

***Corresponding Author**:

Tim M. Bruns
Department of Biomedical Engineering
University of Michigan
2800 Plymouth Road
NCRC B10-A169
Ann Arbor, MI, 48109, USA

Table S1: Table of cats and analyzed trials. Data reported as: total trials (# of sessions).

| Cat/Agent | Alfaxalone | Propofol | Dexmedetomidine | Isoflurane | α-chloralose |
|---|---|---|---|---|---|
| Cat #1 | 8 (3) | 9 (3) | 11 (3) | 3 (1) | 2 (1) |
| Cat #2 | 11 (4) | 11 (4) | 9 (3) | 3 (1) | 3 (1) |
| Cat #3 | 10 (3) | 10 (3) | 9 (3) | N/A | N/A |
| Cat #4 | 10 (3) | 9 (3) | 9 (3) | 3 (1) | 3 (1) |
| Cat #5 | 12 (3) | 9 (3) | 10 (3) | 3 (1) | 3 (1) |

Table S2: Least squares means estimates and 95% confidence intervals for CMG parameters.

| | Alfaxalone | α-chloralose | Dexmedetomidine | Isoflurane | Propofol |
|---|---|---|---|---|---|
| BLADDER CAPACITY (ml) | | | | | |
| Estimate | 46.72 | 59.61 | 42.93 | 54.54 | 44.18 |
| Std Error | 8.56 | 9.18 | 8.56 | 9.11 | 8.56 |
| Lower 95% | 12.11 | 29.49 | 8.38 | 24.10 | 9.71 |
| Upper 95% | 81.33 | 89.74 | 77.48 | 84.97 | 78.65 |
| ΔPRESSURE (cm $H_2O$) | | | | | |
| Estimate | 97.98 | 108.08 | 101.53 | 84.24 | 116.53 |
| Std Error | 9.53 | 13.51 | 9.61 | 13.15 | 9.61 |
| Lower 95% | 74.78 | 80.15 | 78.33 | 56.91 | 93.32 |
| Upper 95% | 121.17 | 136.01 | 124.74 | 111.57 | 139.74 |
| COMPLIANCE (ml/cm $H_2O$) | | | | | |
| Estimate | 0.64 | 0.58 | 0.48 | 0.61 | 0.49 |
| Std Error | 0.12 | 0.16 | 0.12 | 0.15 | 0.12 |
| Lower 95% | 0.33 | 0.24 | 0.18 | 0.28 | 0.18 |
| Upper 95% | 0.95 | 0.91 | 0.79 | 0.94 | 0.80 |
| NVC (# of NVC/s) | | | | | |
| Estimate | 0.012 | 0.033 | 0.002 | 0.008 | 0.010 |
| Std Error | 0.003 | 0.005 | 0.003 | 0.005 | 0.003 |
| Lower 95% | 0.006 | 0.023 | -0.004 | -0.002 | 0.003 |
| Upper 95% | 0.019 | 0.043 | 0.009 | 0.017 | 0.016 |
| NVC AMPLITUDE (cm $H_2O$) | | | | | |
| Estimate | 6.93 | 7.93 | 5.42 | 7.05 | 4.94 |
| Std Error | 0.99 | 1.41 | 1.03 | 1.48 | 0.99 |
| Lower 95% | 4.56 | 5.02 | 3.03 | 4.03 | 2.57 |
| Upper 95% | 9.30 | 10.84 | 7.81 | 10.07 | 7.31 |
| SLOPE 1: start to T-100 (cm $H_2O$/s) | | | | | |
| Estimate | 0.03 | 0.03 | 0.02 | 0.03 | 0.06 |
| Std Error | 0.01 | 0.01 | 0.01 | 0.01 | 0.01 |
| Lower 95% | 0.00 | 0.00 | 0.00 | 0.01 | 0.04 |
| Upper 95% | 0.05 | 0.06 | 0.05 | 0.06 | 0.09 |
| SLOPE 2: T-100 to T-50 (cm $H_2O$/s) | | | | | |
| Estimate | 0.27 | 0.51 | 0.17 | 0.28 | 0.36 |
| Std Error | 0.05 | 0.08 | 0.05 | 0.08 | 0.05 |
| Lower 95% | 0.16 | 0.34 | 0.06 | 0.12 | 0.24 |
| Upper 95% | 0.39 | 0.67 | 0.29 | 0.43 | 0.47 |
| SLOPE 3: T-50 to void (cm $H_2O$/s) | | | | | |
| Estimate | 1.10 | 0.63 | 1.45 | 0.57 | 0.65 |
| Std Error | 0.11 | 0.18 | 0.11 | 0.17 | 0.11 |

| | | | | | |
|---|---|---|---|---|---|
| Lower 95% | 0.85 | 0.27 | 1.19 | 0.23 | 0.40 |
| Upper 95% | 1.36 | 0.98 | 1.70 | 0.92 | 0.91 |

Table S3: p values of Tukey's HSD all pairwise comparisons for each CMG parameter. Significance: * $p < 0.05$, ** $p < 0.01$.

| Agent 1 – Agent 2 | | p values | | | | | | | |
|---|---|---|---|---|---|---|---|---|---|
| Agent 1 | Agent 2 | Bladder capacity | ΔPressure | Compliance | NVC (#/s) | NVC amplitude | Slope 1 | Slope 2 | Slope 3 |
| Alfax | Chloralose | 0.0182* | 0.9138 | 0.9868 | 0.0003** | 0.9356 | 0.9987 | 0.0295* | 0.0449* |
| Alfax | Dex | 0.5598 | 0.9875 | 0.2117 | 0.0084** | 0.3635 | 0.9999 | 0.2091 | 0.0094** |
| Alfax | Iso | 0.2943 | 0.7521 | 0.9995 | 0.8543 | 1.0000 | 0.9522 | 1.0000 | 0.0129* |
| Alfax | Propofol | 0.8520 | 0.0749 | 0.2538 | 0.8941 | 0.0881 | <.0001** | 0.4156 | 0.0002** |
| Chloralose | Dex | 0.0008** | 0.9820 | 0.9379 | <.0001** | 0.3123 | 0.9967 | 0.0004** | <.0001** |
| Chloralose | Iso | 0.8612 | 0.4920 | 0.9993 | 0.0004** | 0.9847 | 0.9973 | 0.1376 | 0.9990 |
| Chloralose | Propofol | 0.0026** | 0.9547 | 0.9531 | <.0001** | 0.1411 | 0.0724 | 0.3240 | 0.9999 |
| Dex | Iso | 0.0338* | 0.5628 | 0.8055 | 0.8115 | 0.7596 | 0.9321 | 0.6539 | <.0001** |
| Dex | Propofol | 0.9873 | 0.2378 | 1.0000 | 0.1137 | 0.9773 | <.0001** | 0.0018** | <.0001** |
| Iso | Propofol | 0.0790 | 0.0449* | 0.8371 | 0.9925 | 0.5194 | 0.1567 | 0.8371 | 0.9886 |

Table S4: Least squares means estimates and 95% confidence intervals for anesthetic parameters.

| | Alfaxalone | α-chloralose | Dexmedetomidine | Isoflurane | Propofol |
|---|---|---|---|---|---|
| HEART RATE (beats per minute) | | | | | |
| Estimate | 215.16 | 163.29 | 110.66 | 130.73 | 176.72 |
| Std Error | 5.69 | 9.49 | 5.75 | 8.18 | 5.77 |
| Lower 95% | 201.38 | 144.13 | 96.87 | 113.89 | 162.92 |
| Upper 95% | 228.94 | 182.44 | 124.45 | 147.58 | 190.52 |
| ΔHR (beats per minute) | | | | | |
| Estimate | 45.36 | | 13.79 | | 37.50 |
| Std Error | 6.65 | | 6.80 | | 7.05 |
| Lower 95% | 27.42 | | -4.01 | | 19.81 |
| Upper 95% | 63.31 | | 31.59 | | 55.19 |
| Time to lateral recumbency (min) | | | | | |
| Estimate | 9.49 | | 5.00 | | 2.42 |
| Std Error | 1.49 | | 1.54 | | 1.62 |
| Lower 95% | 6.41 | | 1.85 | | -0.92 |
| Upper 95% | 12.56 | | 8.15 | | 5.76 |
| Time to head up (min) | | | | | |
| Estimate | 37.24 | | 1.73 | | 43.22 |
| Std Error | 6.10 | | 6.07 | | 6.28 |
| Lower 95% | 24.10 | | -11.33 | | 29.81 |
| Upper 95% | 50.38 | | 14.80 | | 56.64 |
| Time to walking (min) | | | | | |
| Estimate | 55.26 | | 2.53 | | 73.70 |
| Std Error | 10.64 | | 10.58 | | 10.64 |
| Lower 95% | 32.72 | | -19.86 | | 51.16 |
| Upper 95% | 77.80 | | 24.92 | | 96.24 |

Table S5: p values of Tukey's HSD all pairwise comparisons for each anesthetic parameter. Not all parameters were assessed for terminal agents (isoflurane and α-chloralose). Significance: * $p < 0.05$, ** $p < 0.01$.

| Agent 1 – Agent 2 | | p values | | | | |
|---|---|---|---|---|---|---|
| Agent 1 | Agent 2 | HR | Δ HR | Time to lateral recumbency | Time to head up | Time to walking |
| Alfax | Chloralose | <.0001* | | | | |
| Alfax | Dex | <.0001* | <.0001* | 0.1206 | <.0001* | 0.0009* |
| Alfax | Iso | <.0001* | | | | |
| Alfax | Propofol | <.0001* | 0.7766 | 0.0093* | 0.7039 | 0.3585 |
| Chloralose | Dex | <.0001* | | | | |
| Chloralose | Iso | 0.0187* | | | | |
| Chloralose | Propofol | 0.5539 | | | | |
| Dex | Iso | 0.0561 | | | | |
| Dex | Propofol | <.0001* | 0.0082* | 0.5086 | <.0001* | <.0001* |
| Iso | Propofol | <.0001* | | | | |

Table S6: p values of Tukey's HSD pairwise comparisons for reflexes during non-terminal trials. Significance: * p < 0.05, ** p < 0.01.

| Anesthetic agents | | p value | | | |
|---|---|---|---|---|---|
| Agent 1 | Agent 2 | Jaw Tone | Withdrawal | Palpebral | Pupil dilation |
| Alfaxalone | Dexmedetomidine | 0.0003* | <.0001* | <.0001* | <.0001* |
| Alfaxalone | Propofol | 0.0753 | 0.9604 | 0.8070 | 0.4890 |
| Dexmedetomidine | Propofol | 0.1965 | <.0001* | <.0001* | <.0001* |

Table S7: p values of Steel-Dwass pairwise comparisons for effects of animal, session, and trial number of session on urodynamic and anesthetic parameters. Data shown as “level – level (p-value)”. NSF = no significant findings between any pairwise comparisons. Only significant ($p > 0.05$) pairwise comparisons are shown, * $p < 0.05$, ** $p < 0.01$. ΔHR data only available for animals 3-5.

| | By Animal (1-5) | By Session (1-9) | By Trial number of session (1-3) |
|---|---|---|---|
| Δpressure (cm $H_2O$) | 4-5 (0.0094) **<br>2-1 (0.0297) *<br>5-3 (0.0489) *<br>3-2 (0.0093) **<br>4-2 (0.0061) ** | NSF | 3-1 (0.0077) ** |
| Bladder Capacity (ml) | 4-1 (<0.0001) **<br>4-3 (0.0007) **<br>4-2 (0.0009) **<br>5-1 (<0.0001) **<br>5-3 (<0.0001) **<br>5-2 (<0.0001) **<br>5-4 (<0.0001) ** | NSF | NSF |
| Compliance (ml/cm $H_2O$) | 4-2 (<0.0001) **<br>4-3 (0.0018) **<br>4-1 (0.0212) *<br>5-1 (0.0004) **<br>5-3 (<0.0001) **<br>5-2 (<0.0001) **<br>5-4 (<0.0001) ** | NSF | NSF |
| NVCs (NVCs/s) | 4-1 (0.0342) * | 9-6 (0.0122) *<br>9-1 (0.0270) *<br>9-3 (0.0314) * | NSF |
| Slope 1 (cm $H_2O$/s) | 5-4 (0.0049) **<br>4-3 (0.0063) ** | NSF | 2-1 (0.0097) **<br>3-1 (0.0039) ** |
| Slope 2 (cm $H_2O$/s) | 5-1 (0.0016) **<br>5-4 (0.0071) **<br>2-1 (0.0051) **<br>5-3 (0.0340) ** | NSF | NSF |
| Slope 3 (cm $H_2O$/s) | 3-2 (0.0370) * | 7-4 (0.0131) *<br>7-2 (0.0436) *<br>9-7 (0.0282) * | NSF |
| Average HR (bpm) | 4-3 (0.0436) * | 7-2 (0.0362) *<br>6-5 (0.0291) *<br>7-5 (0.0080) ** | NSF |
| ΔHR (bpm) | 5-4 (0.0016) ** | NSF | NSF |

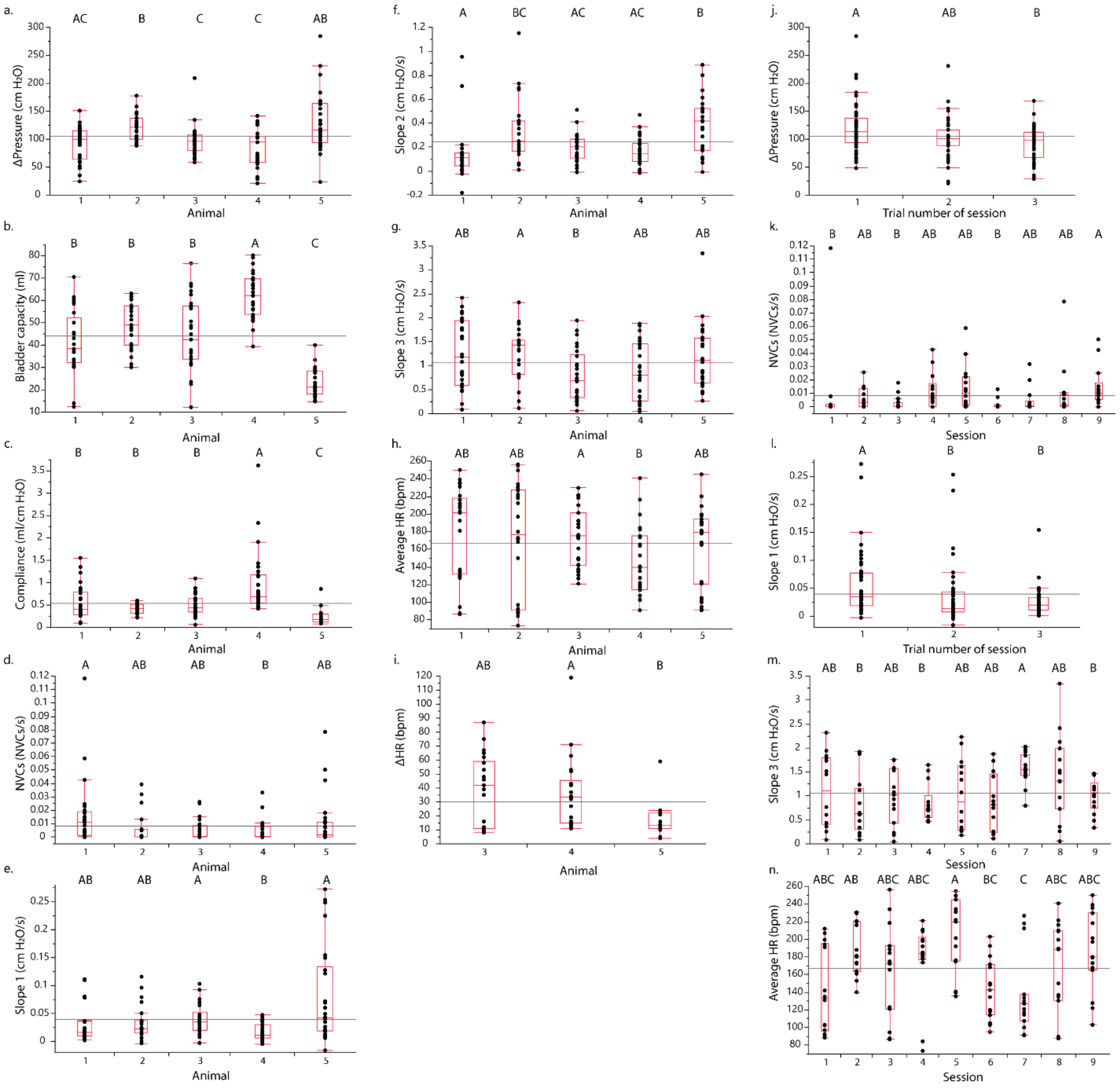

Figure S1: Means of CMG and anesthetic parameters that varied by animal (a-i), session (k, m, n), or trial number of session (j, l). Gray horizontal lines represent the mean of the variable on the y-axis. Boxes represent 1st to 3rd quartile range (interquartile range [IQR]), with the line in the middle of each box representing the median. Tips of whiskers extending below and above box represent 1st quartile – 1.5*IQR and 3rd quartile + 1.5*IQR respectively. Different letters represent significant differences at $p < 0.05$.

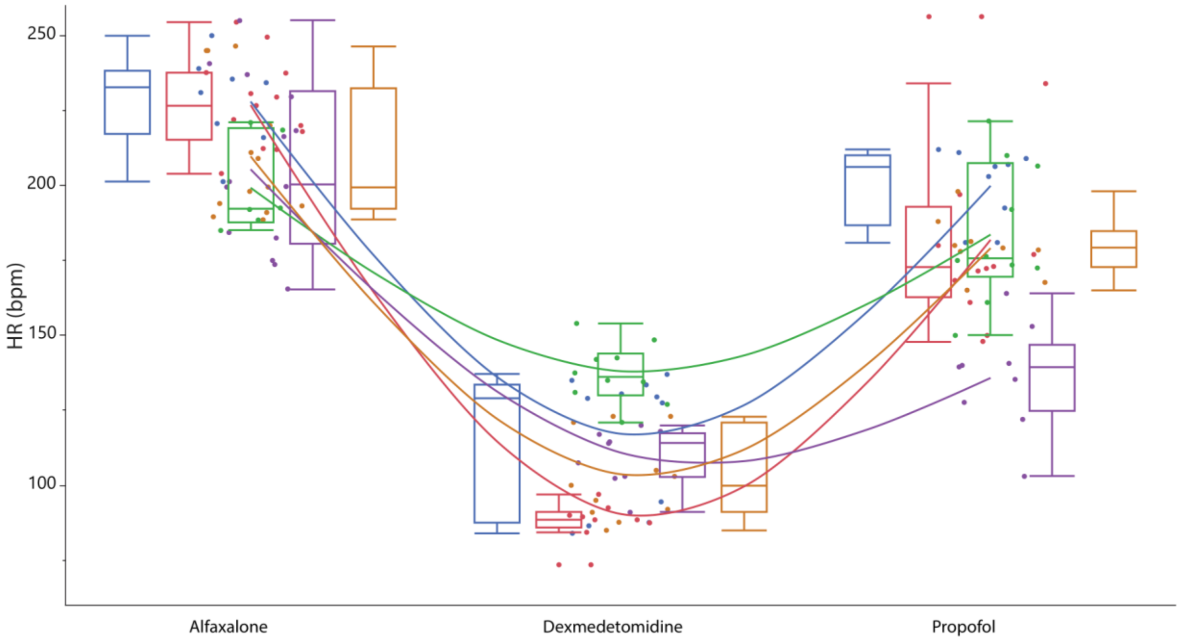


Figure S2: Random jitter scatter plots and box plots of average HR of survival agents, separated by animal. Each color is an individual animal, from left to right for each agent. Boxes represent 1st to 3rd quartile range (interquartile range [IQR]), with the line in the middle of each box representing the median. Tips of whiskers extending below and above box represent 1st quartile – 1.5*IQR and 3rd quartile + 1.5*IQR respectively. The curves are cubic splines that are overlaid to show the per-animal trends.

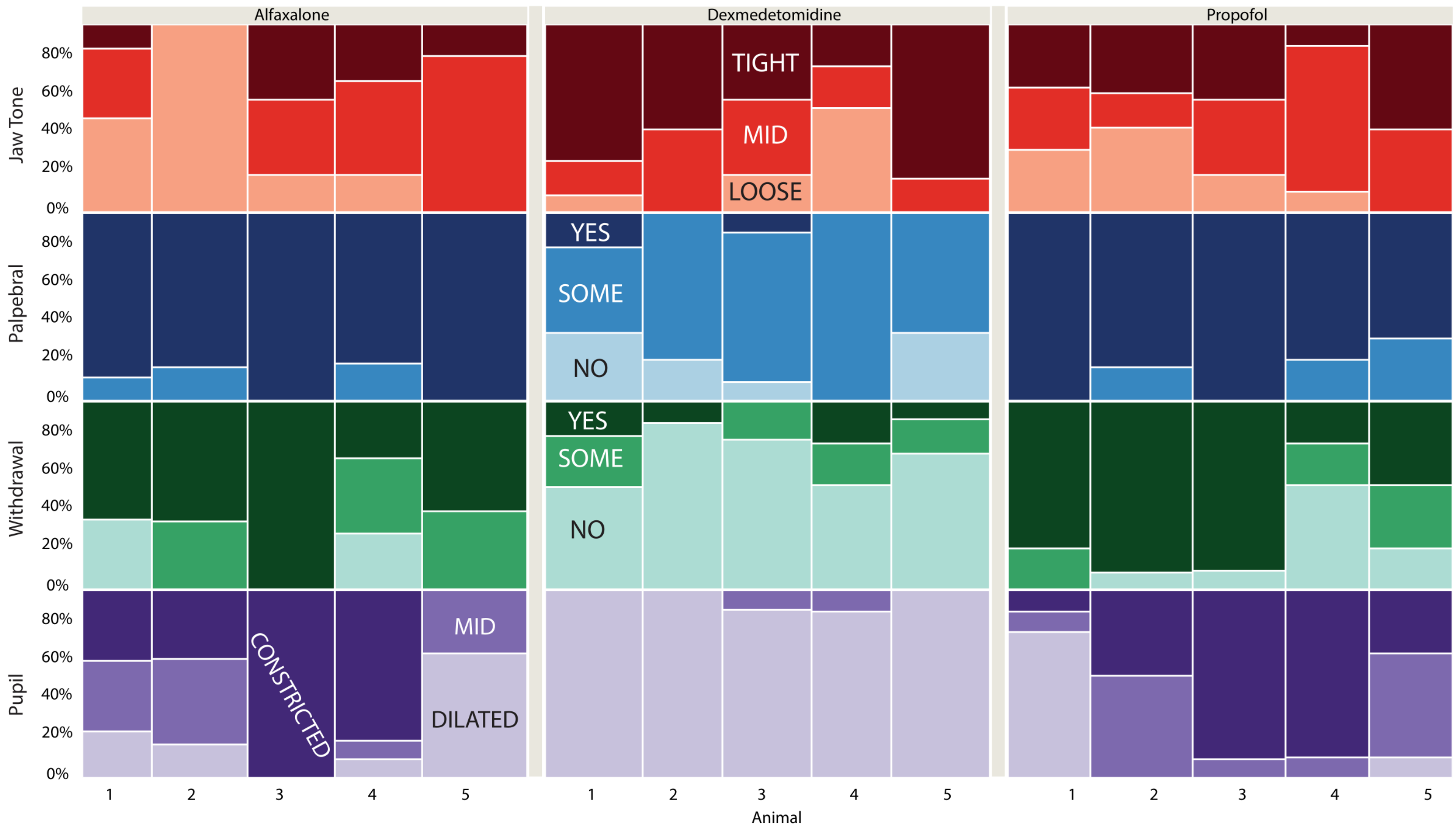


Figure S3: Reflex distribution by survival agents across all trials separated by individual animal. Darker colors indicate a lighter plane of anesthesia
